# Time poverty: Obstacle to women’s human rights, health and sustainable development

**DOI:** 10.7189/jogh.10.020313

**Published:** 2020-12

**Authors:** Elizabeth Hyde, Margaret E Greene, Gary L Darmstadt

**Figure:**
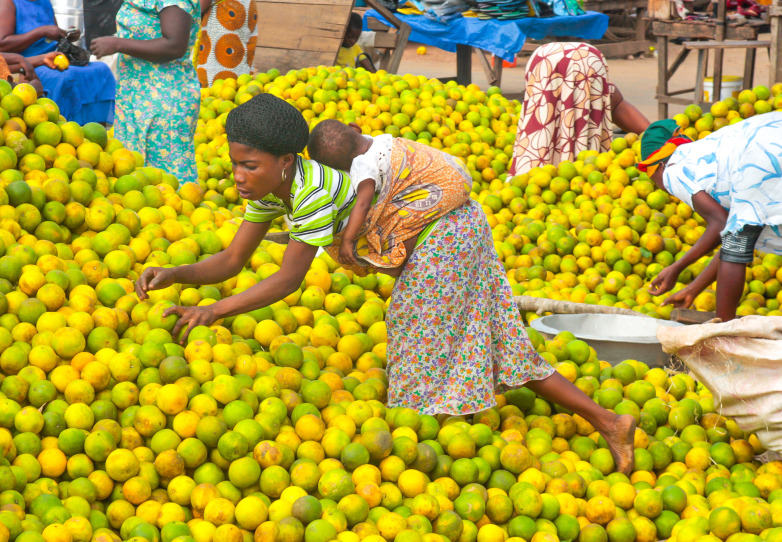
Photo: iStock Getty Images.

Every day, all over the world, women and girls perform countless hours of work without pay. Girls are socialised to be caretakers from an early age. Worldwide, girls between the ages of 10 and 14 spend 50% more time helping around the house than boys of the same age [1]. By adulthood, women in developed and developing nations spend an average of 2 and 3.4 times as many hours per day as men on unpaid work [2], respectively, shouldering the heaviest burden of cooking, cleaning, and caring for children and the elderly. In rural Guinea, for example, women devote an average of 25.6 hours per week to domestic work compared to men's 7.2 hours [3], while in Guatemala, women spend 3.3 hours per day doing unpaid work compared to men's 0.9 hours [4].

Even as female participation in the paid workforce grows, women across widely diverse economies continue to provide the majority of unpaid care work [2]. When all types of work (paid and unpaid) are considered, women work longer days than men on average. In rural Pakistan, 37% of employed women were found to be time poor compared to 19% of employed men because women still carried out domestic responsibilities regardless of employment status [5]. A 2013 survey by the Pew Research Center found that working mothers in the United States spent an average of 14.2 hours per week on housework compared to working fathers' 8.6 hours [6]. That same year, a time-use survey in Mozambique revealed that while women's income-generating work was similar to men's, caregiving and housework were almost entirely women's responsibility [7]. Also in 2013, a study in China found that gender differences in housework-related indicators accounted for 27%-28% of the gender earnings gap [8].

This inequitable gender-based allocation of unpaid domestic work, representing “double-duty” for women who enter the workforce, often leaves women with little or no discretionary time. This is known as time poverty. Time poverty has important repercussions for women's economic opportunities and health, and is a manifestation of the systemic oppression of women via gender inequality and restrictive gender norms which dictate normative expectations for what it means to be male or female in a given society and the roles, responsibilities, and privileges that are allocated to a person based on those norms. Restrictive gender norms limit women's access to paid employment, resources, and control over how resources (including their own time) are used. Time poverty is a human rights issue that must be addressed in order to fulfill the Sustainable Development Goals and empower women and girls everywhere to achieve their full human potential, with lasting benefits for their families, communities and nations.

## HEALTH CONSEQUENCES OF TIME POVERTY

Time poverty harms women's health through numerous pathways (**Table 1**). Domestic responsibilities can leave little time for women to seek medical care, promoting self-neglect. One study in the United States found that almost one-quarter of American women reported delaying or not seeking health care due to insufficient time [9], and another found that being female and having a child in the household to care for were both predictors of delaying HIV care [10]. Among pregnant South African women, daily chores such as fetching water and fieldwork have been shown to decrease use of prenatal care [11].

**Table 1:** The impact of time poverty on women’s health and economic prospects*

**Health:**	
Time poverty promotes self-neglect	• Limited discretionary time due to a large caregiving burden can prevent women from seeking their own medical care [S1]. In 2017, 24% of American women reported delaying or not obtaining health care because they could not find time, and 14% cited trouble finding child care [S2].
	• In a study of HIV patients in the United States, being female and having a child in the household were both predictors of delaying HIV care due to caregiving [S3].
	• Among pregnant South African women, daily chores such as fetching water and fieldwork have been shown to decrease use of prenatal care [S4].
	• Among pregnant women in Benin, educational attainment and being employed – which require sufficient time – were associated with increased utilization of maternal health care [S5].
Time poverty prevents women from earning money, which can limit their ability to pay for health care	• In a study of rural Bangladeshi women, a lack of income-generating activity was associated with increased delay in seeking emergency obstetric care [S6].
Time poverty curtails women's educational opportunities and capabilities for enagaging with health systems	• A study of Aboriginal women in Manitoba found that caregiving responsibilities were a significant barrier to academic progress [S7].
Time poverty results in poorer food choices, less exercise, and more stress	• Time poverty promotes unhealthy eating habits and decreased exercise [8]. Conversely, in a review of the impact of leisure time on health, leisure time was associated with identity formation and affirmation, improved coping during times of stress, and positive effects on work and relationships [S9].
	• A 2017 study found that American fathers engaged in leisure activities 47% and 35% of the time during which mothers did childcare and housework, respectively [S10].
	• Caregiving can also be mentally and physically taxing. Among American women caring for adult relatives, mental health is worse than national norms [S11]. Grandmothers who take significant caregiving roles for their grandchildren have been to shown to suffer increased stress compared to non-caregiving counterparts [S1].
**Economic prospects:**	
Unpaid responsibilities limit women's engagement in the workforce	• Worldwide, three-quarters of men and one half of women are part of the paid labor force [S12]. In 2015, <30% of women in Northern Africa, Western Asia, and Southern Asia worked for pay [S12].
	• Unpaid caregiving duties are a significant barrier to employment, particularly for mothers. In 2013, American mothers were almost three times as likely as fathers to report quitting their jobs at some point for family reasons [S13].
	• Male-dominated occupations often require long hours with little flexibility, which does not accommodate caregiving responsibilities [S14]. Mothers in these fields were 52% more likely to quit than other women if they worked ≥50 hours per week.
	• In the United States, 69% of unpaid caregivers to elderly adults are women [S15]. Daughters and daughters-in-law are more likely than other caregivers to reduce their work hours to care for ageing parents [S16].
Women in the paid workforce are funneled into lower-paid occupations with fewer protections	• Female-dominated professions such as teaching, administrative services, and food production tend to pay less than male-dominated jobs, even when they require the same skill level [S17]. They reflect women’s lower educational attainment, limited mobility, discrimination by employers, normative choices, and the necessity of part-time work to accommodate domestic work [S18]. The same trends are seen in the health sector, where “women care and men cure” [S19].
	• Women tend to occupy lower levels and be paid less than men working in the same industries. For example, in the Canadian food service industry in 2015, 60% of chefs were male, while 72% of kitchen helpers were female [S20]. Women are under-represented in high-paid sectors like technical and business services [S18,S21,S22].
	• Lower-paid roles tend to offer poorer working conditions and be excluded from social protection programs designed to reduce social and economic vulnerability [S22,S23,S24].
	• In the Middle East, legal coverage for employment injury is 18 percentage points lower for women than overall coverage rates [S25].
Gender segregation in the workplace persists due to overt and subtle harassment and discrimination	• The decline in occupational segregation by gender in the United States has significantly slowed in recent decades, regardless of the education level required for the work [S26]. In 2018, only 7.2% of American women worked full-time in male-dominated (≥75% male) fields [S27].
	• Male-dominated occupations are often hostile environments for women and have the highest rates of gender-based harassment [S28,S29]. Women majoring in majority-male fields face significantly more gender harassment than women in other majors [S30].
	• Discrimination on hiring and promoting men over women is pervasive in finance and STEM fields, limiting women's advancement and reinforcing gender-based occupational segregation [S29,S31,S32].
	• 37% of women who work mostly with men report that they have been treated as if they were incompetent because of their gender, compared to 18% of women in gender-balanced workplaces [S29].
Women are paid less than men for similar work	• Jobs with more women pay less than those with fewer, even when controlling for education level and skills [S33]. In most countries, across all sectors and occupations, women working full-time earn 70%-90% of what men earn doing the same work [S12].
	• Women earn less than men in all male-dominated occupations and 18 of the 20 most common occupations for women [S27].
	• Devaluation of women's work has been shown to be a primary driver of the gender wage gap [S33]. The overall pay rate of male-dominated occupations in the United States declined as large numbers of women entered the fields between 1950 and 2000 [S33].

*References are presented in Table S1 in the **Online Supplementary Document**.

Without money of their own, women’s inability to afford health care services or medicines may be further exacerbated. In a study of rural Bangladeshi women, not being involved in income-generating activities was associated with increased delay in seeking emergency obstetric care [12].

Time poverty can also result in poorer food choices and less exercise, and can impose significant mental stress on women and girls. One study in the United States found that time-poor individuals are less likely to walk or cycle for exercise, though they are also less likely to purchase fast food [13].

## ECONOMIC CONSEQUENCES OF TIME POVERTY

Time poverty also prevents women from fully engaging in the formal/monetised economy, limited economic productivity and growth in several ways (**Table 1**). Lack of time due to domestic responsibilities impedes women from completing school, obtaining paid work, and working as many hours for pay as men, funneling women into lower-paying jobs. In 2018, women in the United States were five times as likely as men to work in occupations with poverty-level wages across all occupations [14].

This results in a significant loss of income for women and national economies. The World Bank estimated in 2018 that among people aged 25-34 in peak productive and reproductive years, 122 women were living in poverty for every 100 men [15].

Though unpaid care is critical for the functioning of society and the global economy, it is invisible by most metrics of productivity. Though women spend a large fraction of their time working, they are credited with producing only 37% of global GDP [16]. One study in Guatemala estimated that the value of household and caregiving work totaled 30% of the nation's GDP in 2000 [4], yet it remains completely unrecognised in economic statistics.

Increasing the share of income earned by women has been shown to shift spending priorities in favor of future generations. Evidence from diverse settings (eg, Brazil, Bangladesh, Côte d'Ivoire, Mexico, South Africa, United Kingdom) indicates that increasing women's control over resources translates to greater investment in children's nutrition, health, and education. A multi-country study revealed that increasing female representation in parliaments results in greater spending on education as a percentage of GDP [17]. In one analysis of 76 studies conducted in low and middle income countries, women's decision-making power and education were found to correlate strongly with improved outcomes across multiple sectors, demonstrating that expanding women's agency broadly improves health and development for women, their families, and their communities [18].

## CONCLUSION

Time poverty endangers women’s health and the health of their children, sharply limits women’s economic opportunities, and curtails women’s voice and leadership and limits women’s opportunities to exert influence on their societies through their leadership in work and in public life. Time poverty is also a symptom of a more fundamental problem: gendered social norms that give men power over women, assign lesser value to women and their contributions, and maintain the current inequitable distribution of power and wealth between men and women.

Some strides have been made in recent years in recognising the issue and working toward remedies. Efforts have been made to formalise the definition of time poverty], increase men's involvement in unpaid caregiving and housework, implement novel programmes such as financial incentives for low-income women to seek health care when needed, and develop time-saving technologies like stoves that require less gathering of firewood to function.

Improving women's control over their own time benefits their own health and economic opportunities, as well as the health and economic development of their families, communities, nations, and the world. Ultimately, however, women’s time poverty must be addressed as a global moral imperative and a fundamental human right.

## Additional material

Online Supplementary Document
