# Supplementary material for: Time poverty: Obstacle to women’s human rights, health and sustainable development
Source: J Glob Health. 2020 Nov 8;10(2):020313. doi: 10.7189/jogh.10.020313 (PMC7688061; doi:10.7189/jogh.10.020313)
Supplement: Online Supplementary Document [file jogh-10-020313-s001.pdf]

Table S1. References corresponding to the citations in Table 1.

S1 Kim E-J. Caregiver Stress and Related Factors in Korean Households Utilizing Childcare Support by Grandmothers. Soc Work Policy. 10(1):113-129.

S2 Ranji U, Rosenzweig C, Gomez I, Salganicoff A. 2017 Kaiser Women's Health Survey. The Henry J. Kaiser Foundation; 2018.

S3 MD, Crystal S, Cunningham WE, et al. Delays in seeking HIV care due to competing caregiver responsibilities. Am J Public Health. 2000;90(7):1138-1140.

4 McCray T. An issue of culture: the effects of daily activities on prenatal care utilization patterns in rural South Africa. Soc Sci Med. 2004;59(9):1843-1855.

S5 Yaya S, Uthman O, Amouzou A, Ekholuenetale M, Bishwajit G. Inequalities in maternal health care utilization in Benin: A population based cross-sectional study. BMC Pregnancy Childbirth. 2018;18. doi:10.1186/s12884-018-1846-6c

S6 Nahar S, Banu M, Nasreen H. Women-focused development intervention reduces delays in accessing emergency obstetric care in urban slums in Bangladesh: a cross-sectional study. BMC Pregnancy Childbirth. 2011;11(11). https://bmcpregnancychildbirth.biomedcentral.com/articles/10.1186/1471-2393-11-11

S7 Bonnycastle C, Prentice S. Childcare and caregiving: Overlooked barriers for northern post-secondary women learners. Can J Native Stud. 2011;31:1-16.

S8 Kalenkoski C, Hamrick K. How Does Time Poverty Affect Behavior? A Look at Eating and Physical Activity. Appl Econ Perspect Policy. 2013;35(1):89-105.

S9 Mannell R. Leisure, Health and Well-Being. World Leis J. 2007;49(2):114-128.

S10 Kamp Dush CM, Yavorsky JE, Schoppe-Sullivan SJ. What Are Men Doing while Women Perform Extra Unpaid Labor? Leisure and Specialization at the Transitions to Parenthood. Sex Roles. 2018;78(11):715-730. doi:10.1007/s11199-017-0841-0

S11 Caldwell J. Health and Access to Health Care of Female Family Caregivers of Adults With Developmental Disabilities. J Disabil Policy Stud. 2008;19(2):68-79.f

S12 Division UNS. The World's Women 2015, Chapter 4: Work.; 2015. doi:10.18356/9789210573719.

S13 Parker K. Women More than Men Adjust Their Careers for Family Life. Pew Research Center; 2015. https://www.pewresearch.org/fact-tank/2015/10/01/women-more-than-men-adjust-their-careers-for-family-life/

S14 Cha Y. Overwork and the Persistence of Gender Segregation in Occupations. Gend Soc. 2013;27(2):158-184. doi:10.1177/0891243212470510

S15 Johnson R, Wiener J. A Profile of Frail Older Americans and Their Caregivers. Urban Institute; 2006. https://www.urban.org/sites/default/files/publication/42946/311284-A-Profile-of-Frail-Older-Americans-and-Their-Caregivers.PDF

S16 Covinsky K, Eng C, Lui L-Y, et al. Reduced Employment in Caregivers of Frail Elders: Impact of Ethnicity, Patient Clinical Characteristics, and Caregiver Characteristics. J Gerontol. 2001;56(11):M707-M713.

S17 Hegewisch A, Bendick M, Jr, Gault B, Hartmann H. Pathways to Equity: Narrowing the Wage Gap by Improving Women's Access to Good Middle-Skill Jobs.; 2016.

S18 UNDP. Africa Human Development Report 2016: Accelerating Gender Equality and Women's Empowerment in Africa.; 2016.

S19 Hay K, McDougal L, Percival V, Henry S, Klugman J, Wurie H, et al. Disrupting gender norms in health systems: making the case for change. Lancet 2019 Jun 22;393(10190):2535-2549.

S20 Moyser M. Women and Paid Work. Statistics Canada https://www150.statcan.gc.ca/n1/pub/89-503-x/2015001/article/14694-eng.htm

S21 Woetzel, J. et al. The Power of Parity. McKinsey Glob. Inst. Insights Publ. (2015).

S22 World Bank. Gender Equality and Development.; 2012. doi:10.1080/13552070512331332273

S23 The World Bank. Social Protection.

S24 Razavi S, Arza C, Braunstein E. Gendered Impacts of Globalization.; 2012.

S25 ILO. World Social Protection Report 2014-2015: Building Economic Recovery, Inclusive Development and a Social Justice.; 2014.

S26 Blau FD, Brummund P, Liu AY-H. Trends in Occupational Segregation by Gender 1970–2009: Adjusting for the Impact of Changes in the Occupational Coding System. Demography. 2013;50(2):471-492. doi:10.1007/s13524-012-0151-7

S27 Hegewisch A, Tesfaselassie A. The Gender Wage Gap by Occupation 2018. Institute for Women's Policy Research; 2019..

S28 Kabat-Farr D, Cortina L. Sex-Based Harassment in Employment: New Insights into Gender and Context. Law Hum Behav. 2013;38. doi:10.1037/lhb0000045

S29 Parker K. Women in Majority-Male Workplaces Report Higher Rates of Gender Discrimination. Pew Research Center; 2018.

S30 Dresden B, Dresden A, Ridge R, Yamawaki N. No Girls Allowed: Women in Male-Dominated Majors Experience Increased Gender Harassment and Bias. Psychol Rep. 2017;121:003329411773035. doi:10.1177/0033294117730357

S31 Seron C, Silbey S, Cech E, Rubineau B. Persistence Is Cultural: Professional Socialization and the Reproduction of Sex Segregation. Work Occup. 2015;43. doi:10.1177/0730888415618728

S32 Blair-Loy Mary. It’s not just what you know, it’s who you know: technical knowledge, rainmaking, and gender among finance executives. In: Steven Vallas, ed. The Transformation of Work. Vol 10. Research in the Sociology of Work. Emerald Group Publishing Limited; 2001:51-83. doi:10.1016/S0277-2833(01)80021-2

S33 Levanon A, England P, Allison P. Occupational Feminization and Pay: Assessing Causal Dynamics Using 1950–2000 U.S. Census Data. Soc Forces. 2009;88(2):865-891. doi:10.1353/sof.0.0264
